# Design Space and QbD Approach for Production of Drug Nanocrystals by Wet Media Milling Techniques

**DOI:** 10.3390/pharmaceutics10030104

**Published:** 2018-07-25

**Authors:** Leena Peltonen

**Affiliations:** Division of Pharmaceutical Chemistry and Technology, Drug Research Program, Faculty of Pharmacy, University of Helsinki, P.O. Box 56, 00014 Helsinki, Finland; leena.peltonen@helsinki.fi; Tel.: +358-50-448-0726

**Keywords:** drug nanocrystals, wet media milling, Quality by Design (QbD), Critical Quality Attributes (CQA), process variables

## Abstract

Drug nanocrystals are nanosized solid drug particles, the most important application of which is the improvement of solubility properties of poorly soluble drug materials. Drug nanocrystals can be produced by many different techniques, but the mostly used are different kinds of media milling techniques; in milling, particle size of bulk sized drug material is decreased, with the aid of milling beads, to nanometer scale. Utilization of Quality by Design, QbD, approach in nanomilling improves the process-understanding of the system, and recently, the number of studies using the QbD approach in nanomilling has increased. In the QbD approach, the quality is built into the products and processes throughout the whole production chain. Definition of Critical Quality Attributes, CQAs, determines the targeted final product properties. CQAs are confirmed by setting Critical Process Parameters, CPPs, which include both process parameters but also input variables, like stabilizer amount or the solid state form of the drug. Finally, Design Space determines the limits in which CPPs should be in order to reach CQAs. This review discusses the milling process and process variables, CPPs, their impact on product properties, CQAs and challenges of the QbD approach in nanomilling studies.

## 1. Introduction

Drug nanocrystals are carrier-free nanoparticles: solid drug nanosized particles surrounded by a stabilizer layer [1,2,3,4]. Typically, their size is around a couple of hundred nanometers. The most important application of drug nanocrystals is for improved solubility of poorly soluble drug materials; nanosizing increases tremendously the interfacial area taking part in dissolution, and according to the Noyes–Whitney equation, the interfacial area is straightly correlated to dissolution rate [5,6]. However, drug nanocrystals also have applications in controlled drug release or in targeted drug delivery [7,8,9,10,11].

Production of drug nanocrystals can be divided into two different approaches: (i) top-down and (ii) bottom-up techniques [5,12,13]. In top-down methods, particle size is decreased from coarse drug particles to nanoparticles, for example, by different kinds of wet milling or high-pressure homogenization techniques [14,15,16,17]. In bottom-up techniques, drug nanocrystals are built molecule by molecule, for example, by anti-solvent precipitation or different kinds of liquid atomization-based techniques [7,18,19,20,21]. Combination of individual techniques can be used in order to improve the process efficiency, like reaching smaller particle sizes, or to avoid clocking of the equipment [22,23,24].

When nanosuspension formulations are produced, the process needs to be optimized case-by-case after the preliminary screening studies by studying the effects of critical process parameters on the target product profile with the aid of factorial design. Instead of measuring product and process qualities, the Quality by Design, QbD, approach aims to build quality into products and processes throughout the whole manufacturing chain [25,26,27]. The QbD approach is also supported by the authorities. In the QbD approach for drug nanocrystals, first stabilizer(s) and manufacturing process are selected, then Critical Quality Attributes, CQAs, are defined and finally, design space is formed [28].

In this review, the QbD approach for nanomilling of pharmaceuticals is presented. In order to understand the critical process parameters, milling process and different kinds of mill designs are shortly described, after which the most important process parameters with case studies are discussed. Terminologies related to different steps of the QbD approach are explained and finally, case studies highlighting the different steps of the QbD approach in nanomilling are described on a practical level. 

## 2. Milling Process

**Figure 1:**
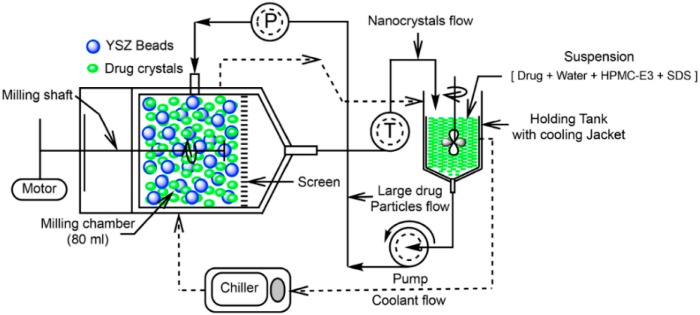
An example of preparation of drug nanocrystals in a recirculation model with hydroxypropyl methylcellulose (HPMC) and sodium dodecyl soleplate (SDS) as stabilizers. YSZ: yttrium-stabilized zirconium. Reprinted from [35] by Creative Commons Attribution 4.0 International Public License.

Nanomilling is the most utilized technique for production of drug nanocrystals, and most commercial products are produced by some kind of milling technique [4,12]. As a process, milling is an old technique that has been utilized a lot, especially, for example, in milling of minerals, in mining and in painting engineering [29]. In pharmaceutics, nanomilling has been studied intensively since the early 1990s [30,31,32,33,34]. The wide utilization of milling is based on considerably easy scale-up and good repeatability of the technique. A schematic presentation of the nanomilling process is presented in Figure 1.

In nanomilling of pharmaceuticals, size reduction is performed in the liquid suspension form. The coarse aqueous suspension of the drug and stabilizer(s) is fed into the mill together with hard beads as milling media. The mechanical attrition of drug particles with the milling media decreases particle size of the drug material. Typically, zirconium oxide (often yttrium stabilized) or polystyrene beads are used as milling media [36,37,38,39,40]. Hard ceramic materials minimize wearing of the beads/equipment. The wear of beads is related to the structure and hardness of the beads, but also to the hardness and shape of the milled particles [41]. By operating in an optimal energy input area with qualified material, wear from grinding media can be minimized [39]; for example, intensification of the wet stirred media milling process by a faster process and lower energy consumption has been shown to decrease the wearing of yttrium-stabilized zirconium oxide beads [40].

During the milling process, two competing processes take place: particle breakage and aggregation [42]. Particle breakage is due to mechanical stress, and aggregation is due to attractive inter-particle forces (for example van der Waals, hydrophobic-hydrophilic forces). At the same time, a lot of particles are stressed, and if the amount of milling media (number of beads) is high, it can be assumed that due to lack of free motion volume, most of the stress events are pressure stressing [43].

Size reduction in media milling is dependent on number and intensity of collisions between the drug particles and milling beads [33,44]. In nanomilling, drug crystals are colliding with the milling media, with the drug itself, and with the milling chamber [45]. Most of the breakage forces in media milling are due to drug particle–bead interactions. In jet-milling and high pressure homogenization techniques, both the particle–particle and particle–wall interactions impact the breakage forces [46], but in the media milling of drug materials, the roles of particle–particle interactions are minor.

Ostwald ripening may take place if the solubility differences between different particle sizes leads to transport of material from smaller to larger particle surfaces [37]. Particle size of the product is dependent on process–equipment parameters and properties of drug material. After the milling process, physical stability of the milled nanocrystal suspension, e.g., stability against aggregation and Ostwald ripening is dependent on the efficiency of the selected stabilizer. The key parameter for stable nanosuspension is selection of stabilizer(s) type and concentration.

Another stability issue with drug nanocrystals is the solid state stability of the drug material. Formation of amorphous material or polymorphic changes may take place during production of nanosystems [47,48]. In bottom-up techniques, especially in liquid atomization-based techniques, solidification of drug in amorphous form is quite common. In nanomilling, presence of water stabilizes the crystalline state, but polymorphic changes are possible [49].

### 2.1. Stabilizers

In the milling process, selection of stabilizer(s) is the crucial step for successful formation of drug nanocrystals, and this is also the first step in the QbD approach [2,13,27,50,51,52]. The stabilizer stabilizes newly formed nanosized particle surfaces towards aggregation in order to maintain the small particle size. The importance of stabilizer selection has been demonstrated in numerous studies, like in a study by Ghosh et al. [53], where selection of stabilizer was found to be the most significant factor affecting particle size, followed by milling speed, drug amount and bead size. In the literature, there are studies trying to rationale the stabilizer selection, but still the selection is mostly made on a trial and error basis [16,34,54].

One stabilizer can be enough for stabilizing drug nanocrystals [14], but often two stabilizers, typically a mixture of a polymer, steric stabilizer, like hydroxypropyl methyl cellulose, HPMC, and an ionic surfactant, electrostatic stabilizer, like sodium dodecyl sulphate, SDS, are used in order to further improve the stabilization effect [16,50,53,55]. Steric stabilization is recommended if possible electrolysis of ionic stabilizers in the gut are supposed to reduce stabilizing efficiency [56]. Examples of different stabilizer–drug combinations are shown in Table 1.

When polymeric stabilizers are utilized, also the molecular weight is an important factor [54]. Liu et al. studied stabilization efficiency of five different polyoxyethylene/polyoxypropylene, PEO/PPO, block co-polymers [54]. In interaction studies, they found differences in the binding efficiencies of different studied polymers. However, the most critical for efficient stabilization was the length and structure of the hydrophilic chain of the polymer, which was forming the steric stabilization layer on top of the particles. If the chain length was too short, the stabilization effect was not good. Polymeric stabilizers can also affect milling efficiency via increasing the viscosity, and this is extremely important, when the milling design is changed, for example in during scale-up, because viscosity effect can be altered during scale up changes [16,53].

After stabilizer(s) selection, the stabilizer(s) amount needs to be decided, and stabilizer amount is often one variable in factorial design [64,65]. The amount of stabilizer should always be studied case by case, but steric stabilizers are used approximately from 10:1 to 1:0.8 (*w*/*w* drug:stabilizer) ratios and surface active stabilizers from 20:1 to 1:0.5 (*w*/*w* drug:stabilizer) ratios.

### 2.2. Process Parameters in Milling

**Figure 2:**
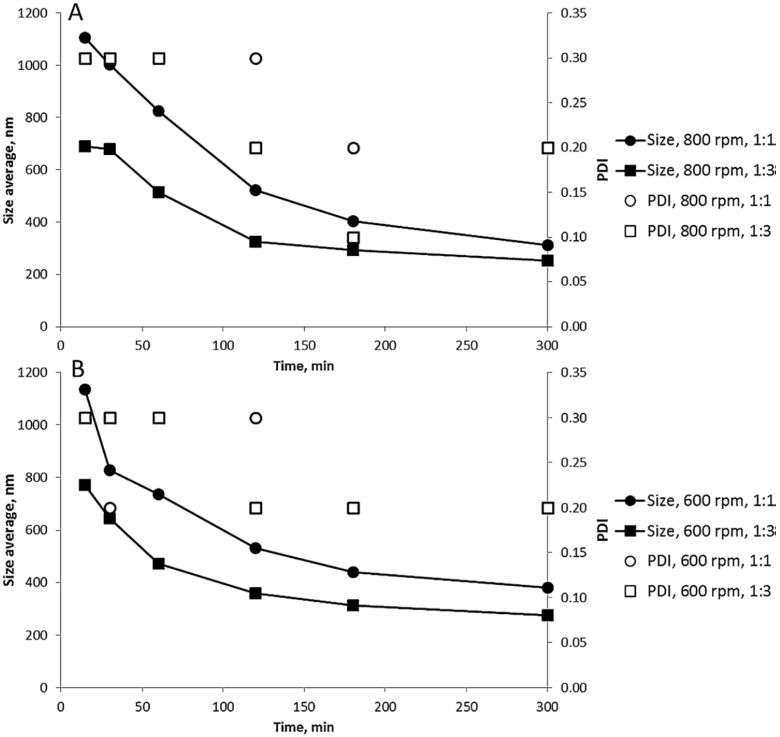
Effect of milling time, milling speed, and bead amount (suspension:bead ratio) on average particle size and polydispersity index of dexamethasone nanocrystals. Bead size: (**A**) 0.1 mm; (**B**) 0.3 mm. Reprinted from [63] with permission from Elsevier.

In the milling process, mechanical energy (specific energy input) is utilized in order to break down coarse drug particles into smaller ones. The complexity of the milling process was demonstrated in a study where 44 different parameters having impact on the milling were recognized [66]. In pharmaceutical nanomilling, most of the parameters have minor effects; the most important parameters having the greatest impact on end-product properties are milling time, milling medium (most important bead size and amount of milling medium, e.g., amount of beads), milling speed, drug amount and milling design (Figure 2) [32,67]. The liquid dispersion medium, typically water in nanomilling, is also introduced as a process parameter in media milling, but so far, its impact on product properties has rarely been studied [68].

Milling time and milling speed are not scientifically exact measures, and their comparison between different milling designs is difficult. Together, they determine the energy input to the system during the milling, and instead, it is recommendable to use the energy input value [33,69]. In continuous milling equipment, the exact determination of milling time is difficult, when processing time for different individual particles can vary. However, in many pharmaceutical studies, milling time and milling speed have been used as milling parameters, and for that reason, those parameters are also discussed in this review. On the other hand, the amount of milling media (number of beads), bead size and drug loading determine the collision intensity, e.g., frequency of compressions, and contact pressure, and these two parameters are also discussed together in the following sections. 

In milling, the minimum average final particle size which can be reached with selected process parameters (energy input) is constant and the particle size is closing this value as a function of time [32,50,67] (Figure 2). During the process, the relative importance of the process parameters can also be altered. The particle size is decreased during the process, and the importance of process parameters can be related to the particle size of the milled material. For example, when the final particle sizes of the end-product were 5 μm, 2 μm, 1 μm, 500 nm and 200 nm, rotational speed was mostly affecting the particle breakage with the largest particles, while grinding media size has the biggest impact in breakage of smallest particle size fractions [70]. Colombo et al. [63] studied the effect of milling time, milling speed and amount of milling beads on the average particle size and polydispersity index of formed dexamethasone nanocrystals (Figure 2). Higher amount of beads increased the milling efficiency and smaller particle sizes were reached faster. Changing the milling speed from 600 rpm to 800 rpm had only a minor effect on the product properties, as well as changing the pearl size from 0.1 mm to 0.3 mm.

#### 2.2.1. Frequency of Compressions and Contact Pressure

Bead size is an important process parameter [35,38,40,67], and the common rule is that smaller beads lead to smaller nanocrystals, though this is not always true, like the case for example with highly viscous suspensions [44,69]. For example, Li et al. used yttrium-stabilized zirconia beads with sizes of 50–800 µm [40]. They found out that only beads with 50–100 µm in size led to sub-100 nm griseofulvin particles.

Typically, contamination risk is also higher with larger beads [28,40,71]; when the above-mentioned griseofulvin particles were milled with 50 µm beads, the process time was shortest and also the bead contamination and specific energy consumption was lowest [40]. With the smallest beads, the frequency of drug particle compressions was highest and bead contact pressure was lowest, which lowered the level of contamination. Also, high milling speed increases contamination risk [28].

Depending on the suspension properties, beads with different sizes can act differently. Medarevic et al. used beads with diameters of 0.1 mm, 0.5 mm and 1.0 mm [72]. The amount of polymer, hydroxypropyl cellulose (stabilizer), and milling speed were the other variables. When beads with 0.1 mm diameter were used, particle size decrease was very efficient with low polymer concentration and low milling speed. With higher polymer concentration, high milling speed was needed due to increased viscosity of the suspension. Smaller and lighter beads did not have enough kinetic energy in more viscous suspensions if the milling speed was low, but, with the larger beads (0.5 and 1 mm), lower milling speed and lower polymer concentration did not lead to efficient formation of nanocrystals: smaller particles were reached only with high stabilizer concentration and high milling speed.

The impact of individual variables can often be difficult to separate. When 0.1 mm and 0.5 mm milling beads were compared in a study by Ghosh et al., milling with smaller beads resulted in smaller particles with 400 rpm milling speed [65], but when milling speed was lowered to 150 rpm, larger beads resulted smaller particles. Normally specific energy input combined to smaller bead size results smaller particle size. But, when the specific energy input is reduced to very low level, the larger beads are able to compensate the energy loss, and in that case larger beads results finer end-product.

Higher drug amount in the milling vessel means higher collision frequency of the particles. Too low drug amount can produce higher contamination due to the wearing of the vessel and beads. When the effect of drug loading on the bead–bead collisions and particle breakage kinetics was studied, higher drug amounts led to slower breakage [69]. Related to the drug loading during the milling, high concentration may increase the aggregation tendency of the newly formed nanoparticles.

Narayan et al. [73] studied the effect of milling time, bead size, stabilizer concentration and drug amount on particle size and polydispersity of the suspension. Particle size was decreased with longer milling time and smaller milling beads. Increased polymer concentration (polyvinyl alcohol, PVA) increased the particle size due to higher viscosity. Higher drug amount also increased the particle size. The effect of all the variables was lower to the polydispersity value of the suspension. The larger milling pearls, higher amount of PVA, and higher drug amount increased the polydispersity of the suspension, while longer milling times decreased it.

Nakach et al. noticed also with higher drug loadings a significant decrease in milling kinetics, which was explained by the changes in the viscosity of suspension due to the higher drug amount [74]. When three different process parameters (milling speed, bead size, drug amount) were studied, the effect of milling speed was two times higher on the final particle size as compared to the total drug amount [65]. In almost all the studied compositions, higher drug amount led to smaller final particle size. Reason for this was that higher solid content increased the attrition between the solid particles.

#### 2.2.2. Energy Input

In the 1990s, it was typical that nanomilling lasted hours or even days. Nowadays, more and more high energy mills are utilized and milling times for production of good quality drug nanocrystals can be as short as only a couple of minutes [14,75]. Longer milling time increases contamination risk [40,70]. When the effects of milling time and milling speed on the particle size of the final product were studied, both variables were having significant impact [76].

**Figure 3:**
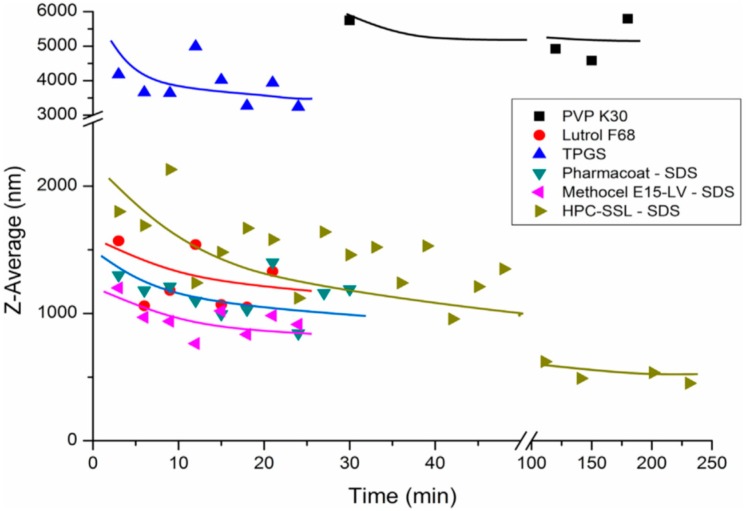
Effect of milling time on determined particle size for different stabilizers studied. Polydispersity index is below 0.5 in most of the batches. Reprinted from [75] with permission from Elsevier.

Generally, longer milling time is expected to facilitate comminution due to the higher number of collisions between drug particles and milling beads [75], but, long milling may increase the temperature in the milling vessel, which can increase crystal growth due to higher tendency for Ostwald ripening in the system. Higher temperature may also be problematic, if the drug material has low melting temperature, like for example ibuprofen. Mechanical properties of the crystals can be altered, which affects the fracturing kinetics. Aprepitant nanocrystals required a considerably long milling time to reach homogenous small particle sizes in a study made by Toziopoulou et al. (Figure 3) [75]. This is due to the fact that aprepitant crystals are anisotropic in mechanical properties. In aprepitant crystal, (001) crystal face is more resistant to fracturing, and its relative surface area is much higher, as compared to more fragile (100) crystal face. Hence, most of the collisions are facing harder (001) crystal faces, which lowers the milling efficiency.

Too long milling times can also induce small particle aggregation [65]. Additional mechanical energy can break the repulsive forces between the already formed nanocrystals, which results in the formation of aggregates. Accordingly, care must be taken when particle sizes are analyzed. If individual particles are aggregating, particle size analysis can give misleading information about the particle breakage kinetics of the nanomilling process.

The milling time has shortened dramatically during the years and utilization of high energy mills have increased milling speeds. However, depending on milling design, given milling speeds are not comparable between different studies. Ghosh et al. studied milling speed, size of milling beads and drug amount as process parameters by a Quality by Design (QbD) approach using three different polymeric stabilizers (hydroxypropyl methylcellulose, HPMC; polyvinylpyrrolidone, PVP; hydroxypropyl cellulose, HPC) with Vitamin E TPGS as a surface active agent [53]. The outcome was that milling speed was the most significant process parameter influencing particle size.

In the same study, upscaling from the planetary ball mill (25 mL batch size) to lab scale stirred media mill (5 L batch size) was performed [53]. Scaling up was successful and particle size results were comparable. However, in the stirred media mill the milling speed effect on particle size with HPMC as a stabilizer was negative (contradictory behavior as compared to planetary ball mill). This was explained to be caused by differences in agitation principle between planetary and stirred media mills; the influence of viscosity changes was different depending on the mill design. In a study by Singare et al., polymer (stabilizer) concentration as well as the milling speed was noticed to affect significantly the zeta potential value of the particles [76]. Changes in zeta potential value can have an impact on the long term stability of the nanosuspension, if the stabilization relies on electrostatic stabilization. 

#### 2.2.3. Milling Design

As already discussed in earlier sections, milling design can affect the relative importance of different process parameters. Milling can be performed for example by a stirred media mill [53], a planetary ball mill [14], or agitation can be induced for example via a magnetic stirrer [77], orbital shaker [59] or acoustic mixing [50], and different milling set-ups affect breakage kinetics and milling efficiency differently. This is seen for example when making scale-up or scale-down changes [78]. The milling behavior of a certain mill is characterized by the stress event intensity, and the stress energy connected to each of the stress event [79]. Based on this, a comminution result is determined by the number of stress events, and stress intensity. The product of stress number and stress energy is proportional to the specific energy input. The proportionality factor is the energy transfer factor, which describes the percentage of energy that is used for stressing the product particles. Using the characteristic numbers based on a few tests, the operating parameters can be optimized and different mills can be compared.

When scaling up is performed, new important formulation variables and process parameters, which may have an impact on the nanosuspension production at larger scales, can be found [76,78]. For example, industrial scale requirements like high polymer concentration and high milling speed may increase production temperature and pressure on the milling equipment, which may require modulations in the milling speed and time. Milling can also be started with lower speed followed by a slow increase in speed. It is important to notice that often preliminary screening studies before a systematic QbD approach are performed in small scale systems, for example in 96-well plates, where again the milling design is totally different [59].

Ghosh et al. studied the effect of differences in agitation in different milling design by comparing small scale rotating planetary mill and lab scale impeller mill [65]. It was found that a planetary mill with 400 rpm produced similar particle sizes as impeller mill with 2500 rpm. When particle size data from these two mills after process optimization were compared, no significant differences were found. This study demonstrated the need to understand properly the scale up induced milling efficiency changes and also, the impact of milling design on the milling efficiency; in different setups, milling speeds are not comparable and the relation between the different setups needs to be found.

Yuminoki et al. used a rotation/revolution mixer for scaling the media milling process from 100 mg of drug to 1 kg [80,81]. Theoretical specific collisional energy was calculated by an equation, which was modified for a wet milling processes. Based on calculations, the relative centrifugal acceleration of revolution (corresponds to radius of the revolution and the number of revolutions per minutes) and drug loading in the suspension where two most important process variables. If these factors where kept constant, different scalings produced similar particle size fractions.

In the previous sections, impact of different process parameters has been described with case studies. Table 2 summarizes the typical effect of different parameters on final product properties. However, the table should be interpreted with care, because often several process parameters are affecting simultaneously, and the interpretation of the role of one single parameter is not always clear. 

## 3. Quality by Design, QbD, Concept

The Quality by Design, QbD, concept is a global regulatory initiative for guiding pharmaceutical development regarding the direction of proactive design of pharmaceutical manufacturing processes and controls in order to build the quality and intended performance of a product inside the product all the time throughout the manufacturing process [25,26,82,83]. QbD relevant pharmaceutical development principles are presented in the ICH guidance documents (ICHQ8-11).

QbD emphasizes better product know-how and process understanding throughout the manufacturing chain. Process understanding should be based on scientific level research/analysis combined with risk management. Mathematical modeling can be helpful and it is integral part of QbD approach throughout the product lifecycle in various parts of the QbD chain: determination of the design space, control strategy and risk management [84]. With the aid of QbD, for example, new technologies can be adapted faster to be part of the manufacturing processes. Economic benefits are gained via reduced number of failed batches, and shorter reaction times towards possible failures. Most importantly, the final aim all the time is safe and efficacious end-product for the customer, which increases the patient compliance.

Mathematical models in QbD are mostly used for determining the design space [84], but also for control strategy analysis. Models present in a simplified way system or phenomena with the aid of mathematical terms. Models can be mechanistic-based on only physical laws, for example, in the case of nanomilling an energy balance. Another possibility is to use existing data for the modeling, for example, with the aid of multivariate analysis/chemometrics; in that case model is called empirical model. Also hybrid models, combining mechanistic and empirical models, can be used. 

### 3.1. QbD Approach for Nanosuspension Production

For drug nanosuspensions, QbD approach can be divided into three steps: (i) stabilizer(s) and manufacturing process(es) selection; (ii) definition of Critical Quality Attributes, CQAs; and (iii) formation of Design Space [28].

**Figure 4:**
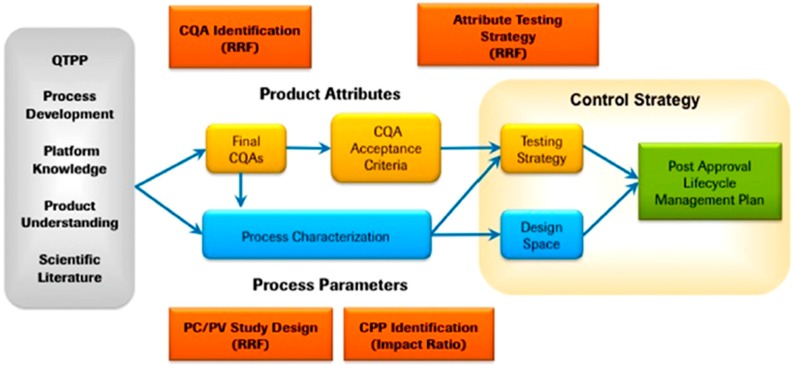
Example of Quality by Design process steps in coordinated decision-making framework for manufacturing of biopharmaceuticals. PC/PV = process characterization and process validation; RRF = risk ranking and filtering tool. Reprinted from [82] with permission from Elsevier.

In the first step, for the stabilizer(s) and process selection, Quality Target Product Profile, QTPP, should be determined carefully. In step two, for drug nanosuspensions particle size and size deviation, shape, solubility/dissolution or stability are typical examples of CQAs [85]. The formation of a Design Space is based on Design of Experiments (DoE). Proper recognition of Critical Process Parameters, CPPs, enable efficient process controlling by finding suitable process control tools throughout the production. Well-defined Design Space results in repeatable product performance from batch to batch. One case example of what could be different process steps for QbD approach in pharmaceutics is presented schematically in Figure 4.

When starting the nanomilling tests, preliminary optimization of the set-up needs to be done by varying only one parameter at a time and keeping the other parameters constant. In this preliminary phase, the CPPs affecting selected CQAs are recognized [86]. In order to study more precisely the impact of certain variables, design setups can be utilized; the 2^2^ factorial design set-up was utilized for studying the effect of concentration levels of two different stabilizers [87], 3^2^ factorial design to study the effect of stabilizer concentration and antisolvent–solvent ratio on particle size, drug content and 90% drug release time [88], 3^2^ factorial design to study the effect of amount of stabilizer and process time [89], or Box–Behnken design was used for studying the effect of drug, polymer and surfactant concentrations and milling time [77] or polymer and surfactant concentrations and milling time and speed and the final product properties determined were particle size distribution, zeta-potential and scalability of the process [76]. Utilization of design setups can minimize the number of tests to be performed but maximize the information gained.

From preliminary studies, the knowledgebase for reasonable classification of process parameters, based on their criticality for the system in question, can be determined. Also, the acceptable variation ranges for the operation are based on preliminary studies. Design Space is determined based on the process and product information reached in the preliminary studies. Accordingly, the careful planning of the preliminary tests is extremely important, because all the decisions for the QbD approach in the beginning of the process are based on the information from those studies. Besides, data from these studies is the bases for defining the effect the production processes can have on the fluctuation of CQAs. Testing and monitoring strategy is defined based on this information, and all the parameters should be controlled and, if necessary, redefined throughout the product life cycle. In the following sections, QbD parameters are presented more profoundly.

#### 3.1.1. Quality Target Product Profile, QTPP

QTPPs are the quality characteristics of the final product, which will be reached in order to confirm the desired quality, safety and efficacy of the final product. Production of nanocrystals by nanomilling is just a first step of the process towards the final drug product. Accordingly, for example, when making stabilizer selection, it is important to keep in mind the final product properties, not just the properties of drug nanocrystals. For example, stabilizer selection should not only be based on the stabilizing effect, but stabilizers can function as permeation enhancers or maintaining the supersaturated state after the dissolution in the final product [4,90]. One example of possible QTPPs, when the final dosage form is ocular suspension, are particle size and polydispersity, sterility, viscosity, isotonicity and stability. Determination of QTPPs requires deep understanding of the relations between end-product quality attributes and their effect on safety and efficacy. After determination of end-product QTPPs, CQAs can be defined. Examples of possible TPPs for drug nanocrystals are presented in Table 3.

#### 3.1.2. Determination of Critical Quality Attributes, CQAs

CQAs are physical, chemical or microbiological properties, which should be inside a determined limit in order to ensure the quality of the product. For example, in the case of drug nanocrystals, particle size, particle size deviation, particle shape, zeta potential or solid state form of the drug can be CQAs depending on the final product properties and drug delivery route [91,92,93]. Determination of CQAs starts by listing all the known quality attributes. This is based on the preliminary characterization of the nanosuspensions after the screening studies. At the same time, a thorough literature survey should be done in order to identify potential other attributes. The next step is to recognize reliably the critical attributes from the list, which often is very laborious. Reevaluation is advisable to be done also later, when level of knowledge from the studied systems is increased [25,83]. Examples of CQAs for drug nanocrystals and how they are affected by different process variables (Critical Material Attributes, CMAs and CPPs) are presented in Table 4.

In a study by Karakucuk et al., critical quality attribute for a nanosuspension, which was aimed for iv administration in a preclinical study, was determined to be microbiological purity of the suspension [94]. Nanosuspension was produced in small scale by ball milling in glass vials and the milling time was 2 h. It was shown in the study that the energy produced during the nanomilling process was sufficient to destroy all kind of microorganisms. The critical process parameter was found to be milling time; at least 2 h milling time was required for reaching the predetermined microbiological purity level. No bacterial growth was noticed in the suspension during 14 days of incubation after the milling. 

#### 3.1.3. Defining CPPs

CPPs are process parameters, which control CQAs. They are monitored during the process in order to confirm the quality of the final product. Individual CPPs do not necessarily impact all CQAs and on the other hand more than one CPP is typically affecting one CQA. Accordingly, CPPs and their impact on CQAs, should be evaluated both individually and in interaction with each other; here, modeling like multivariate analysis techniques are helping the process characterization. In nanomilling, for example, bead side diminishing and increased milling speed often have same kind of impact on particle size [64,95].

All the input variables, also critical material attributes, CMAs, can be seen as process parameters, not only the chemical and physical process parameters [25,83]. For example, raw material properties, like crystal form or particle size of the bulk drug or the stabilizer concentration are typical CMAs for drug nanocrystals [61,92,93]. Besides recognition of CPPs, they need appropriate acceptable ranges of operation [83]. These limiting values are based on the process and product knowhow and thorough literature studies and/or screening studies.

#### 3.1.4. Establishment of Design Space

Design Space takes into account multidimensionally the combination and interaction of input variables and process parameters, which are confirming the quality of the end-product. Design Space contains all the unit operations, their process parameters and all the raw materials. The limits of Design Space are set by accepting appropriate limits for each individual dimension (Design Space parameter) of the Design Space [83]. For well-functioning Design Space, impact of process parameters on quality attributes should be well understood. Design Space defines the limits, in which the individual parameters can be altered without causing unwanted changes in the final product properties. It should be noticed that, if occasionally, one dimension of Design Space is outside of the limits, it does not automatically result in unacceptable product quality.

Narayan et al. [73] optimized the production of drug nanocrystals in order to reach minimum particle size with minimum PDI value and maximum zeta potential value. The Design Space determined with the aid of mathematical modeling was based on screening studies. Design Space was wide and the model predicted that nanocrystals with desired properties would be obtained by milling the suspension containing 200 mg of drug in 0.25% PVA-solution for 4 h time with milling beads of 5 mm. Based on the predicted suggestion, laboratory tests were performed, and the CQAs were evaluated to validate the formulation procedure.

All the QbD phases emphasize the importance of hard and reliable data to allow informed decisions to be made. Additional follow up throughout the process time is recommended, especially when the process is fixed, to critically evaluate all the selected parameters [25].

## 4. Discussion

The QbD approach can be seen as an approach where the starting point is affected by the end in mind, meaning that the final product properties, safety and efficacy of the final drug product are considered in the beginning. During the last years, utilization of the QbD approach has been established in nanocrystal studies. Though the milling process itself is extremely complicated, the critical process parameters related to the final targeted product profile are not too many.

When determining CQAs of the final drug formulation, in the case of drug nanocrystals, particle size and size deviation or polydispersity index are important factors. Depending on the drug delivery route, also for example zeta potential, microbiological quality, particle shape or solid state form of the drug can be important. Often, listing of product properties are easier than defining which of them are critical. Also, determination of reasonable acceptance criteria for the CQAs may be complicated.

After the CQAs are decided, CPPs affecting CQAs should be selected. Parameters like milling speed, milling time, bead diameter and amount of beads, stabilizer selection and concentration, drug amount, are examples of process variables. CPPs include both process parameters as well as input variables. Again, listing of process parameters is easier than determining which of them are critical. CPPs and their relative impact on CQAs may change when milling design is changed, for example during scale-up or scale-down. In preliminary studies, it is important to gain enough knowledge from the process parameters that the design space can be decided reliably, and often these screening tests are performed in down-scaled mill setups.

For Design Space, CPPs require acceptance limits. Sometimes the impact of an individual variable may be difficult to recognize, and most of CPPs are interconnected related to their impact on CQAs. QbD process is continuing throughout the process/product lifecycle. It is also important to react regarding the changes in the Design Space, CPPs or CQAs and alter the parameters and requirements accordingly, if new information is gained.

Preliminary screening studies are required for drug nanocrystal studies, but after that, the utilization of the QbD approach should be a standard protocol. Efficient factorial design planning is saving time and money at the same time while one is gaining more qualified research results. 

## Figures and Tables

**Table 1:** Examples of different stabilizers used in screening studies for production of drug nanocrystals.

Stabilizer	Drug	Reference
Methyl cellulose (MC), hydroxypropyl methylcellulose (HPMC), gelatin, sodium alginate, polyvinyl alcohol (PVA), polyvinyl pyrrolidone (PVP), polyethylene glycol (PEG), Tween 80, sodium deoxycholate, Pluronic F68, sodium dodecyl sulphate (SDS), sodium carboxymethyl cellulose (NaCMC), bovine serum albumin	Curcumin	[57]
Whey protein isolate, Poloxamer 188, SDS	Carvedilol	[58]
D-α-tocopherol polyethylene glycol 1000 succinate (TPGS)	Loviride, itraconazole, cinnarizine, indomethacin, mebendazole, naproxen, phenytoin	[59]
SDS, sodium docusate, sodium cholate, Tween 80, Dehyquart^®^ A-CA, Poloxamer 188, TPGS, PVA, HPMC	Amphotericin B, curcumin, hesperetin, ibuprofen, resveratrol, rutoside trihydrate	[60]
PVP, PVA-PEG, Poloxamer 188, TPGS, Tween 80, HPMC, hydroxyethyl cellulose (HEC), hydroxypropyl cellulose (HPC), MC, NaCMC, sodium alginate	Loviride, itraconazole, cinnarizine, griseofulvin, indomethacin, mebendazole, naproxen, phenylbutazone, phenytoin	[61]
Pluronic^®^ F68, Pluronic^®^ 17R4, Tetronic^®^ 908, Tetronic^®^ 1107, Pluronic^®^ L64	Indomethacin	[54]
Plantacare 810 UP, Plantacare 1200 UP, Plantacare 2000 UP	Curcumin	[62]
Poloxamer 407, Poloxamer 188, vitamin E-TPGS, lecithin, Poloxamer 188 + lecithin, Plantacare 2000 UP	Dexamethasone, ibuprofen, tacrolimus	[63]

**Table 2:** Process parameters in milling and their typical impact on final product properties. It should be noticed that normally, several parameters are affecting milling process and the effect of one parameter on the final product properties is not always clear.

Parameter	Comments
Milling time	Commonly longer milling times lead to more monodisperse particles and decreased particle sizes; however, too long milling times may cause aggregation
Milling speed	Higher milling speed typically increase the milling rate and lead smaller particle sizes
Milling medium (bead size and the amount of beads)	Typically smaller beads lead to smaller nanocrystals; higher amount of beads improve the milling efficiency, though too high bead amount increase contamination
Drug amount	Very small drug amount increases contamination and lowers milling efficiency; very high drug amount increases viscosity and decreases milling efficiency
Milling design	Parameters can change a lot between different milling design: important especially in scaling-up/scaling-down

**Table 3:** Example of target product profiles (TPPs) for drug nanocrystals: case aceclofenac. Modified from [73].

TPPs	Target	CQAs
Dosage form	Oral capsule	Identity, assay, content uniformity
Microbial quality	No contamination	Test for microbial growth
Product performance	Robust manufacturing	Drug content, particle size, polydispersity, morphology, solid state form, in vitro drug release
Pharmacokinetic target efficacy and safety	Better pharmacokinetic profile than pure drug	Pharmacokinetic parameters (T_max_, C_max_, AUC, MRT)
Stability	Stable at least 3 months at accelerated storage conditions	Drug content, particle size and polydispersity of redispersed sample

**Table 4:** Example of risk assessment matrix for drug nanocrystals analyzing the impact of critical material attributes (CMAs) and critical process parameters (CPPs) on product attributes: case study for drug nanocrystals. Modified from [73].

CQAs	Risk Assessment Matrix					
	CMAs			CPPs		
	Surfactant Type	Surfactant Concentration	Drug Amount	Milling Time	Milling Speed	Bead Size
Particle size	Medium	Medium	Medium	High	High	High
PDI	Medium	High	Medium	High	High	High
Zeta potential	High	High	Medium	Medium	Medium	Medium
Drug content	Low	Low	Low	low	Low	Low
Drug release	Medium	Medium	Medium	High	High	High
